# RNA-Seq of Cyst Nematode Infestation of Potato (*Solanum tuberosum* L.): A Comparative Transcriptome Analysis of Resistant and Susceptible Cultivars

**DOI:** 10.3390/plants11081008

**Published:** 2022-04-07

**Authors:** Saranya Chandrasekar, Purushothaman Natarajan, Priyank Hanuman Mhatre, Mahesh Mahajan, Sundararaj Nivitha, Venkatasalam E. Palanisamy, Umesh K. Reddy, Palanisamy Sundararaj

**Affiliations:** 1 Department of Zoology, Bharathiar University, Coimbatore 641046, Tamil Nadu, India; sarananitha89@gmail.com; 2 Department of Biology, Gus R. Douglass Institute, West Virginia State University, Charleston, WV 25112, USA; pnatarajan@wvstateu.edu; 3 Department of Genetic Engineering, SRM Institute of Science and Technology, Kattankulathur 603203, Tamil Nadu, India; 4 ICAR-Central Potato Research Institute, Regional Station, Udhagamandalam 643004, Tamil Nadu, India; priyank.iari@gmail.com (P.H.M.); venkat_ep@yahoo.co.in (V.E.P.); 5 Department of Biotechnology, Vilasrao Deshmukh College of Agricultural Biotechnology, Latur 413512, Maharashtra, India; mahimm1@gmail.com; 6 College of Science, Northeastern University, Boston, MA 02115, USA; nivitha.sundar@gmail.com; 7 Plant Germplasm Introduction and Testing Research, USDA-ARS, Pullman, WA 99164, USA

**Keywords:** RNA-seq, potato, cyst nematode, differentially expressed genes, disease resistance

## Abstract

Potato (*Solanum tuberosum* L.) is an important food crop worldwide, and potato cyst nematodes (PCNs) are among the most serious pests. The identification of disease resistance genes and molecular markers for PCN infestation can aid in crop improvement research programs against PCN infestation. In the present study, we used high-throughput RNA sequencing to investigate the comprehensive resistance mechanisms induced by PCN infestation in the resistant cultivar Kufri Swarna and the susceptible cultivar Kufri Jyoti. PCN infestation induced 791 differentially expressed genes in resistant cultivar Kufri Swarna, comprising 438 upregulated and 353 downregulated genes. In susceptible cultivar Kufri Jyoti, 2225 differentially expressed genes were induced, comprising 1247 upregulated and 978 downregulated genes. We identified several disease resistance genes (*KIN*) and transcription factors (*WRKY*, *HMG*, and *MYB*) that were upregulated in resistant Kufri Swarna. The differentially expressed genes from several enriched KEGG pathways, including MAPK signaling, contributed to the disease resistance in Kufri Swarna. Functional network analysis showed that several cell wall biogenesis genes were induced in Kufri Swarna in response to infestation. This is the first study to identify underlying resistance mechanisms against PCN and host interaction in Indian potato varieties.

## 1. Introduction

Potato (*Solanum tuberosum* L.) is well known as “the king of vegetables” and is considered the fourth largest crop in the world [1]. India ranks second in potato production globally, with 48.53 million tonnes produced from 2.15 million ha [2]. As a staple food, potatoes can provide a sustainable food supply and lessen poverty and malnutrition in many parts of the world [3]. Moreover, it can provide starch as a raw material for many potato-based industries, such as for feeding farm animals and as a potential medicine resource [4]. Because of the equinox climate and well-distributed rains throughout the year, the Nilgiri hills of southern India are used to cultivate vast quantities of potatoes [5]. 

Although the productivity of potatoes is high in the Nilgiri hills, the yield potential is threatened by several biotic and abiotic stresses. Infestation with potato cyst nematode (PCN) (*Globodera rostochiensis* and *G. pallida*) is one of the major constraints in potato production worldwide, including in India [6,7,8]. These two species of PCN are differentiated into eight pathotypes (five for *G. rostochiensis*, Ro1 to Ro5, and three for *G. pallida*, Pa1 to Pa3) [2]. Because of their potential impact on plant growth, development, and yield, these species are included in the list of quarantine pests, and the transportation of potatoes is severely affected throughout the globe [9]. Infestation by nematodes severely affects the transportation of water and nutrients from roots due to vascular tissue damage, as well as the quality and quantity of the crop. Moreover, the feeding site (i.e., syncytium) formed by the PCN is metabolically active, acts as a “nutrient sink”, and attracts nutrients produced by plants [10]. In other words, the plant spends major portions of its energy supporting the PCN at the cost of itself, which results in heavy yield losses of up to 80% [11,12]. 

In India, PCN infestation was first reported by F.J.W. Jones, in 1961, in Nilgiri Hills, which led to the implementation of domestic quarantine in 1971 [13]. In 2018, these regulations were extended to three North Indian states—Himachal Pradesh, Jammu Kashmir, and Uttarakhand—and the sale of seed potatoes from these areas was prohibited outside these states because of PCN infestation [14]. Therefore, managing PCN infestation has become a challenge for researchers of sustainable agriculture. However, with the proper blending of different management options (i.e., integrated nematode management), PCN populations can be managed effectively to below the economic injury level [15]. The integrated nematode management approach involves several practices such as trap cropping, rotation with non-host crops, use of nematode-resistant potato varieties, organic amendments to increase the activity of antagonistic microorganisms, and use of biological control agents and nematicides [13,16]. Soil treatment with nematicides is generally not recommended because of the severe health risks to humans, animals, and the environment [17]. To avoid this situation, eco-friendly approaches, such as using resistant varieties, cultural and physical control, and biocontrol agents, etc., are acceptable [10]. However, cultural, physical, and biological control are not efficient because of the several survival adaptations of PCNs, such as a hard protective cyst coat, extended survival in the egg stage for more than two decades, diapause in juveniles, etc. Therefore, the use of host resistance is one of the most sustainable nematode management approaches [2,8,18]. 

Plants harbor specific resistance (R) genes to protect against invading pathogens such as nematodes, viruses, bacteria, insects, and fungi [19]. R genes act as surveillance proteins to protect against specific effector proteins, which are avirulent genes produced by pathogens, in a process called effector-triggered immunity; they recognize pathogen effectors directly or indirectly via sophisticated mechanisms and initiate a series of defense responses [20]. These responses typically include rapid and localized cell death, fast rupture of reactive oxygen species (ROS), induced biosynthesis, accumulation of hormones such as salicylic acid (SA), rapid programmed cell death as a result of a hypersensitive response at the infestation site, and increased expression of pathogenesis-related genes [21,22]. The plant resistance mechanism differs according to the R gene present in host plants and the effector gene products released from oesophageal glands of plant-parasitic nematodes (PPNs) and their interaction with the host species [23].

Hence, understanding how potato cultivars respond to PCN infestation requires a comprehensive evaluation of PCN-induced changes in gene expression. The identification of the plant resistance response will help to open new avenues for PCN management that can be efficiently used in crop improvement programs against PCN infestation. For this, high-throughput RNA sequencing (RNA-seq) has become the foremost choice to measure gene expression [24]. The most common aims of RNA-seq are to identify differentially expressed genes (DEGs) between two or more biological conditions and to infer associated pathways and gene networks from the expression profiles [25]. Several studies demonstrated the use of RNA-seq for comparing resistant and susceptible cultivars with different PPNs [26,27,28,29,30]. 

However, globally, only one study has described the interaction of PCNs with the Swedish potato breeding clone SW-1015 containing the H1 gene. After infestation with the *G. rostochiensis* Ro1/4 pathotype, SW 1015 showed a quick upregulation of many genes, including tomato stress-responsive factor 1, germins, laccase, and cysteine protease, as compared with a susceptible Désirée potato [31]. 

Kufri Swarna is a medium-maturing, high-yield potato cultivar with a high level of PCN resistance suitable for cultivation in the Southern hills of India [32]. It was developed by pollinating a female parent, Kufri Jyoti, with a male parent, (VTn)2 62.33.3. The female parent, Kufri Jyoti, is a high-yield indigenous cultivar with excellent agronomic characteristics. However, the male parent, (VTn)2 62.33.3, is a wild relative of potato belonging to the species *Solanum vernei* that is resistant to PCN. Kufri Swarna was originally released for cultivation in South Indian hills in 1985 as a PCN-resistant cultivar [32]. Recent studies identified multiple R genes/quantitative trait loci (QTLs) (H1, Grp1, GpaVvrn-QTL, and Gpa5-QTL) as the major factors responsible for this resistance. These R genes/QTLs exhibit resistance reactions against different pathotypes of PCN (Ro1,4 of *G. rostochiensis* and Pa2, 3 of *G. pallida*) [2,33]. In India, Kufri Swarna is the most popular and commonly grown PCN-resistant cultivar [33], but the mechanism of resistance is still unknown. 

In the present study, we used RNA-seq to investigate the changes in gene expression underlying the comprehensive resistance mechanisms induced by PCN infestation in the PCN-resistant cultivar Kufri Swarna in comparison to the PCN-susceptible cultivar Kufri Jyoti. 

## 2. Results

### 2.1. Physiological Responses

**Figure 1:**
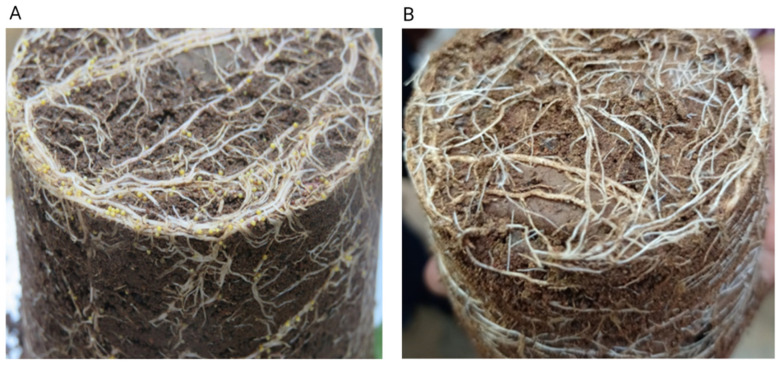
Development of *Globodera* spp. on susceptible Kufri Jyoti (**A**) and resistant Kufri Swarna (**B**) at 55 days after planting in potato-cyst-nematode-infested soil.

By using the root ball technique and observations at 55 days after planting under greenhouse conditions, potting trials demonstrated that cultivar Kufri Jyoti was highly susceptible to PCN (>100 females of *G. rostochiensis* and *G. pallida* per plant), but Kufri Swarna was resistant and supported a minimum number of PCN females (<20 females of *G. rostochiensis* and *G. pallida* per plant) (Figure 1). The formation of a sophisticated nematode feeding site (i.e., syncytium) was observed between 5 and 15 days post-infestation (dpi). The syncytium is the only source of nutrition for developing nematodes (Supplementary Figure S1). The formation of a syncytium was normal in susceptible Kufri Jyoti but weaker in resistant Kufri Swarna at 14 dpi.

### 2.2. RNA-seq and Analysis

**Figure 2:**
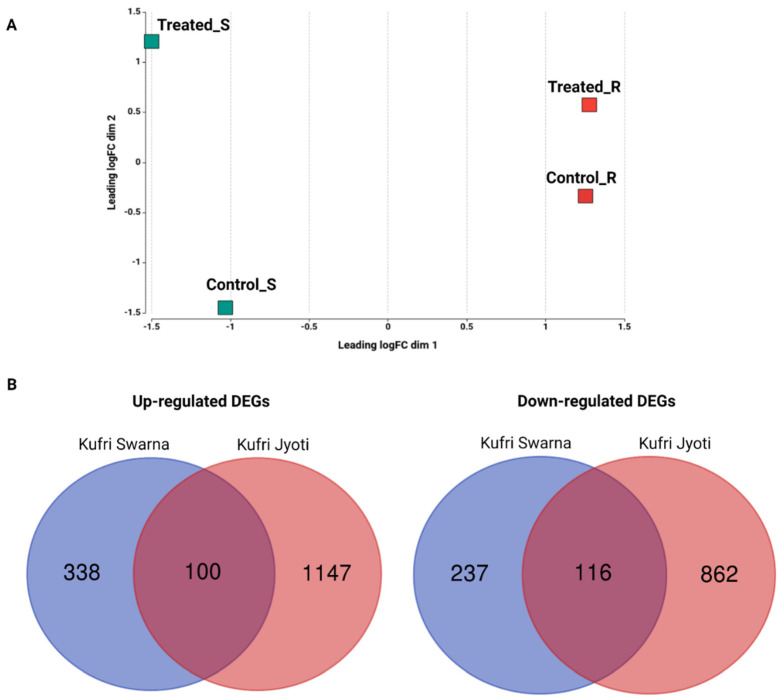
(**A**) MDS plot showing the correlation of expression values among the RNA-seq libraries. (**B**) Venn diagrams showing differentially expressed genes (DEGs) shared between resistant Kufri Swarna and susceptible Kufri Jyoti.

Total RNA was isolated from root tissues of resistant Kufri Swarna and susceptible Kufri Jyoti with the required controls and treatment conditions. An RNA-seq library was prepared for each cultivar by using the total RNA pooled from three biological replicates. The library underwent paired-end sequencing (2 × 75 bp) via the Illumina NextSeq 500 platform. A summary of the RNA sequencing reads is presented in Table 1. The raw RNA sequencing data from the current study were deposited in NCBI’s Short Read Archive database (accession nos. SRX9097233 [inoculated Kufri Jyoti], SRX9097233 [uninoculated Kufri Jyoti], SRX9097231 [inoculated Kufri Swarna], and SRX9097230 [uninoculated Kufri Swarna] under the bioproject PRJNA488526). The raw reads were subjected to stringent quality filtering, and the Q30 percentage of reads in each library was ≥95%. The reads from the two cultivars were aligned to the *S. tuberosum* v6.1 genome [34] by using the STAR universal RNA-seq alignment tool with default parameters. The mapping percentage of the reads per sample ranged from 86% to 90% (Table 1); 2% of the reads remained unmapped. The correlation of the expression among the RNA-seq libraries is presented in Figure 2A.

### 2.3. Differentially Expressed Genes

**Figure 3:**
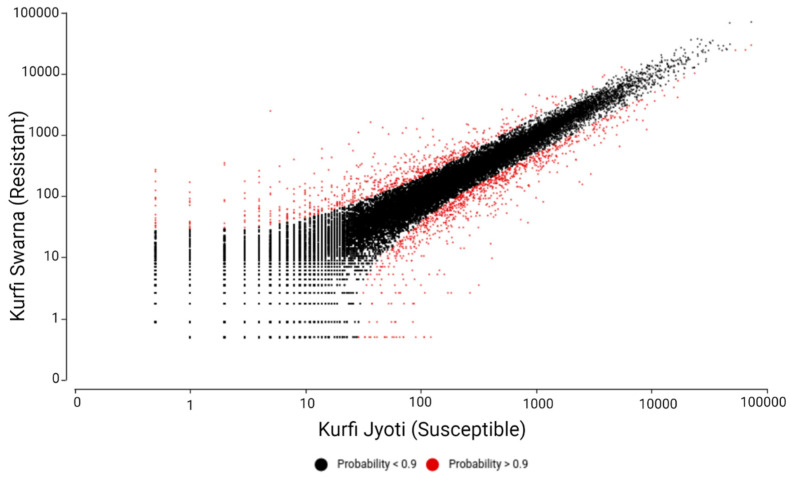
Summary plot of gene expression values for the cultivars Kufri Swarna and Kufri Jyoti. The red points represent genes with expression significant at *p* ≥ 0.9.

The individual read count tables across genes for the two genotypes were created by genome alignment by using the HTSeq R package with TMM normalization. Pair-wise combinations identified DEGs by comparing the resistant and susceptible cultivars with the NOISeq R/Bioc package and three simulated replicates, with variability of 0.02 and a count-per-million value of 1. The DEGs were filtered according to a minimum log2 fold change of 1 and *p*-value of 0.9 as per the NOISeq R/Bioc package. A total of 791 statistically significant DEGs were identified from resistant Kufri Swarna: 438 upregulated and 353 downregulated. Susceptible Kufri Jyoti expressed 2225 DEGs: 1247 upregulated and 978 downregulated. The numbers of genes shared among the up- and downregulated DEGs from the resistant and susceptible cultivars are presented in Figure 2B. The number of DEGs obtained was higher for the susceptible cultivar than for the resistant one, showing increased stress in the susceptible cultivar. The differences in the expression of genes between the resistant and susceptible cultivars are presented in Figure 3.

### 2.4. Pathway Enrichment Analysis

**Figure 4:**
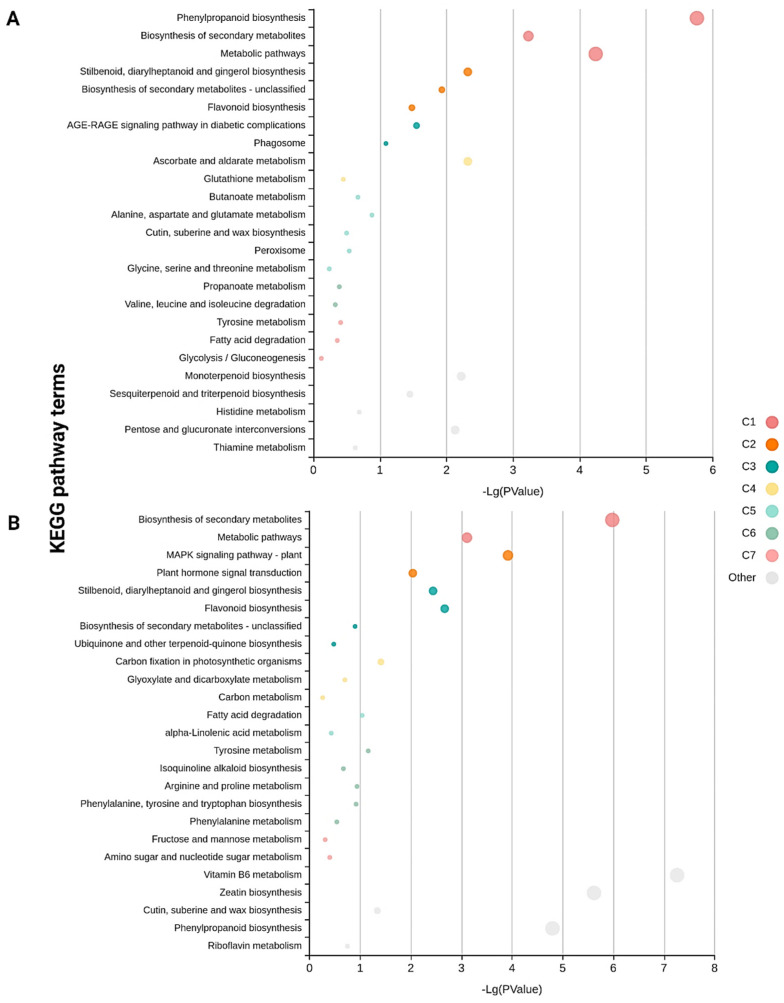
Significantly enriched KEGG pathways among the (**A**) upregulated and (**B**) downregulated DEGs of resistant cultivar Kufri Swarna. The color and size of the dots represent the different functionally enriched clusters and the number of DEGs mapped to the indicated pathways, respectively.

**Figure 5:**
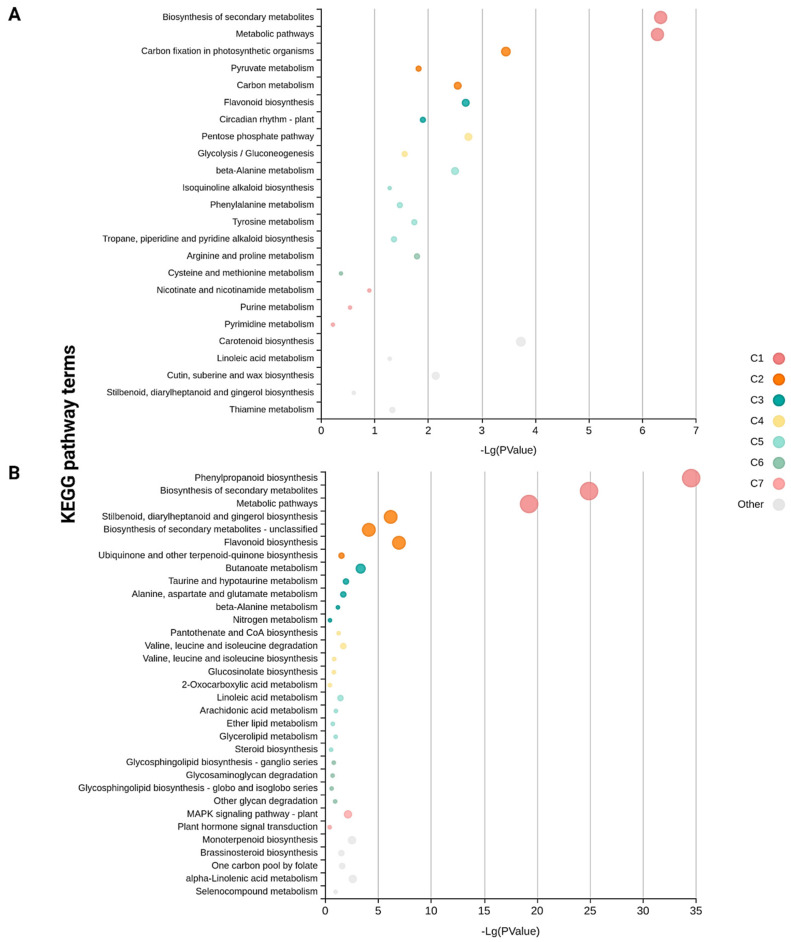
Significantly enriched KEGG pathways among the (**A**) upregulated and (**B**) downregulated DEGs of susceptible cultivar Kufri Jyoti. The color and size of the dots represent the different functionally enriched clusters and the number of DEGs mapped to the indicated pathways, respectively.

Pathway enrichment analysis of the DEGs involved using the Kyoto Encyclopedia of Genes and Genomes (KEGG) pathway database with KOBAS and MapMan. The DEGs from Kufri Swarna and Kufri Jyoti were assigned to 79 and 109 pathways, respectively. The functionally enriched KEGG pathways are in Figure 4 and Figure 5. Mitogen-activated protein kinase (MAPK) signaling and plant hormone signal transduction were enriched in DEGs from resistant Kufri Swarna versus susceptible Kufri Jyoti. Plant MAPK signaling plays an important role in signaling plant defense against pathogen attacks [35]. The cuticle, composed of cutin and wax, plays an important role in plant growth and development and resistance to biotic and abiotic stresses [36]. The cutin, suberin, and wax biosynthesis pathway was enriched in DEGs from Kufri Swarna versus Kufri Jyoti, which is important for disease resistance in plants.

### 2.5. Biotic Stress Pathway

**Figure 6:**
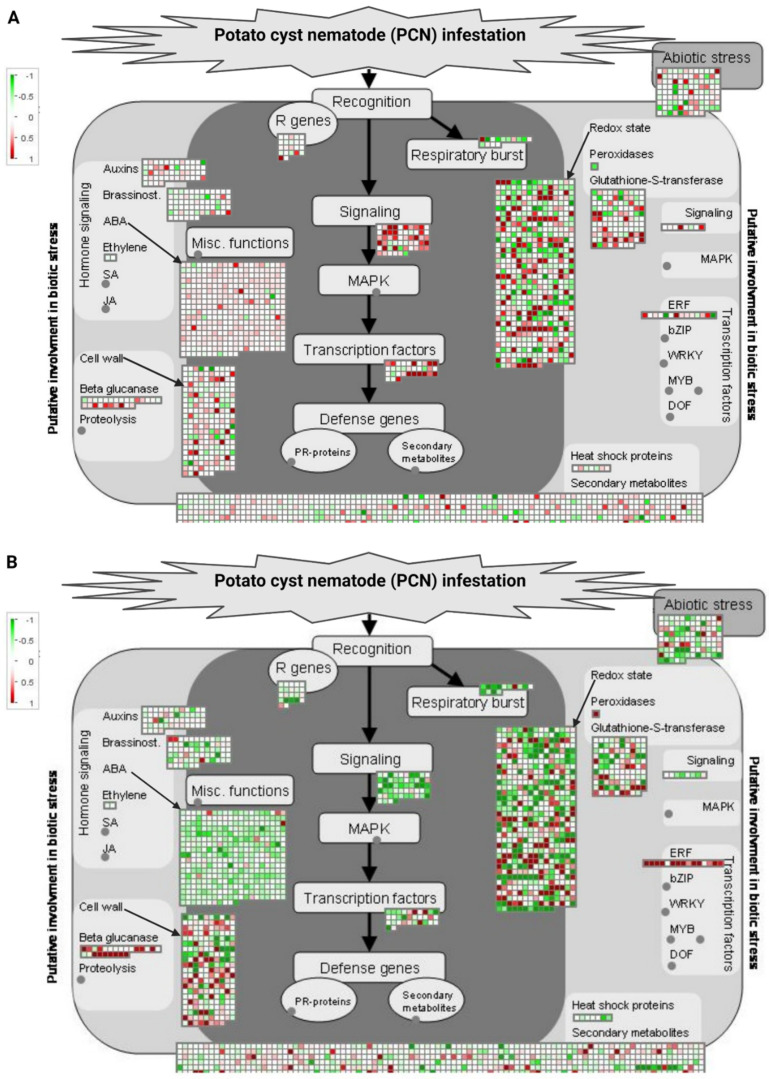
MapMan visualization of DEGs mapped to the biotic stress pathway in resistant Kufri Swarna (**A**) and susceptible Kufri Jyoti (**B**) in response to PCN infestation. DEGs are binned to MapMan functional categories and values are represented as log2 fold change values. Red represents upregulated DEGs and green represents downregulated DEGs. R, response; PR, pathogen-related.

MapMan was used to map the DEGs from the resistant and susceptible cultivars against the biotic stress pathway to show differences in gene expression between the two cultivars in response to PCN infestation. The biotic stress pathway was mediated by genes involved in pathogen recognition, R genes, MAPK signaling, transcription factors (TFs), and defense genes. Plant hormone signaling plays a vital role in controlling biotic stress. On MapMan pathway analysis, many of the important genes involved in the biotic stress pathway for providing disease resistance against pathogens were upregulated in resistant Kufri Swarna versus susceptible Kufri Jyoti (Figure 6). These upregulated genes include effector receptor (NLR), aminotransferase (ALD1), programmed cell death cysteine protease (XCP), defensin (PDF2), RAM1-dependent TF (WRI5), regulatory protein (CBP60) of systemic acquired resistance, LysM receptor kinase (NFR5/NFP), and disease resistance mediator (MLO2/6/12).

### 2.6. DEGs for TFs and Disease Resistance

**Figure 7:**
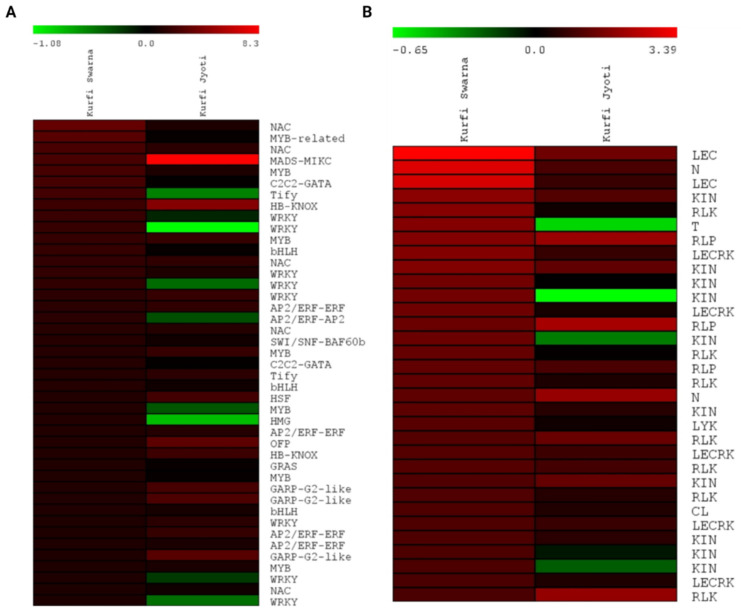
Heatmap showing the expression of DEGs for transcription factors (**A**) and plant disease resistance genes (**B**) in resistant Kufri Swarna and corresponding expression in susceptible Kufri Jyoti in response to PCN infestation. Plant disease-resistance gene classes are included in the gene name. Gene expression values are presented as log2 fold change.

TFs play a major role in regulating genes for pathways relating to disease resistance, biotic stress, and abiotic stress. TFs enriched among the DEGs were analyzed using the Plant Transcription Factor Database (http://planttfdb.cbi.pku.edu.cn/, accessed on 3 February 2022). The DEGs from resistant Kufri Swarna included genes for 23 TF families, mainly including bHLH (10), AP2/ERF-ERF (9), MYB (7), and WRKY (9) TFs. These TFs, playing an important role in disease resistance, were highly upregulated in resistant Kufri Swarna versus susceptible Kufri Jyoti (Figure 7A). Plant R genes encode proteins that initiate pathways leading to biotic-stress-related disease resistance in plants. Overall, 32 R genes were upregulated in resistant Kufri Swarna in response to PCN infestation (Figure 7B); these included KIN (10), RLK (7), LECRK (5), RLP (3), LEC (2), N (2), CL (1), LYK (1), and T (1). Their expression levels are presented in Figure 7B.

### 2.7. Gene Ontology (GO) Enrichment Analysis of DEGs

**Figure 8:**
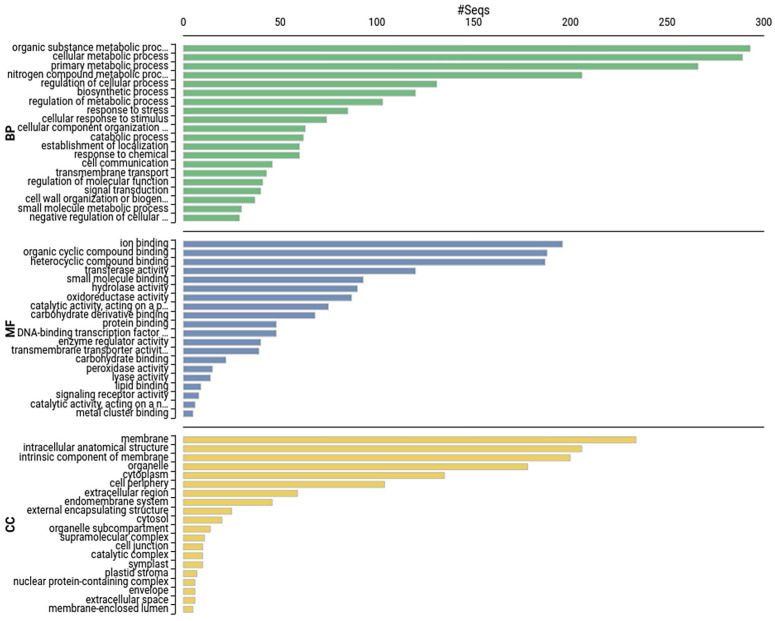
Top 20 terms under the Gene Ontology categories of biological process (BP), molecular function (MF), and cellular components (CC) enriched among the DEGs upregulated in resistant Kufri Swarna.

GO enrichment analysis helps us to understand the molecular and regulatory functions of DEGs under three major categories: biological processes, molecular functions, and cellular components. GO enrichment analysis of DEGs from resistant Kufri Swarna revealed functional categories activated with PCN infestation (Figure 8). The significantly enriched biological processes in resistant Kufri Swarna with PCN infestation included several stress response processes and plant development processes, including response to stress (234 DEGs), cellular response to stimulus (165 DEGs), and signal transduction (101 DEGs).

### 2.8. Functional Network Analysis of DEGs

**Figure 9:**
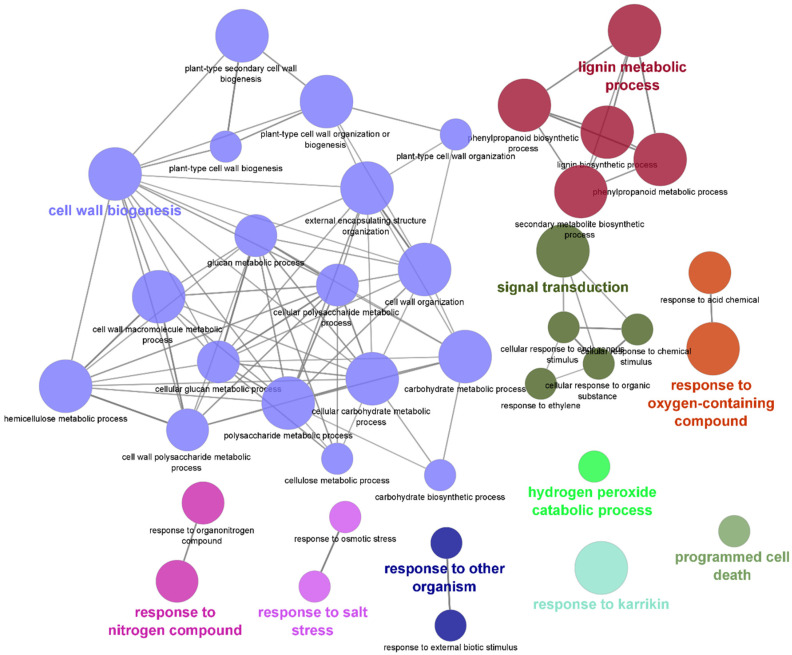
Functional network analysis of DEGs in resistant Kufri Swarna showing the functionally grouped terms with nodes linked based on their kappa score (≥0.3); only the label of the most significant term per group is shown. The node size represents the enrichment significance of the term.

Functional network analysis using Cytoscape and the STRING database revealed the presence of 10 enriched functional network clusters. The enriched functional network categories include cell wall biogenesis, lignin metabolic process, signal transduction, response to oxygen-containing compound, response to nitrogen compound, response to salt stress, response to other organism, and programmed cell death (Figure 9).

### 2.9. Validation of RNA-seq Results with qRT-PCR Analysis

**Figure 10:**
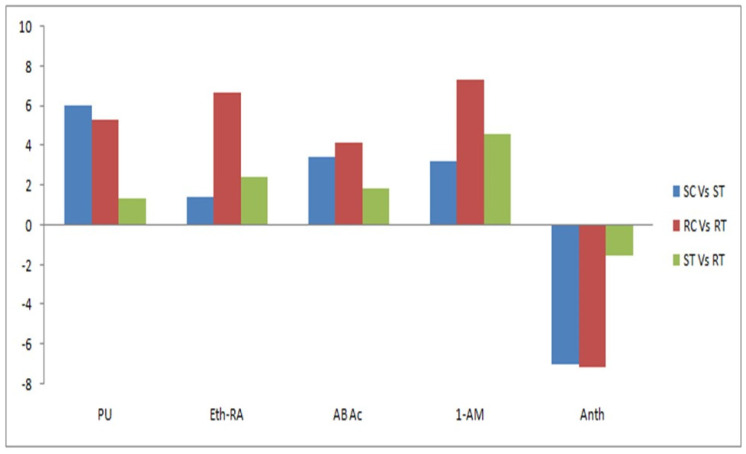
Validation of RNA-seq results with qRT-PCR findings. SC, control susceptible cultivar; ST, treated susceptible cultivar; RC, control resistant cultivar; RT, treated resistant cultivar.

We used quantitative real-time PCR (qRT-PCR) to validate the differential gene expression profile from the RNA-seq results. Five DEGs were selected according to their association with the disease resistance pathway and hormonal signal response. The expression levels of the selected genes determined by qRT-PCR were consistent with the RNA-seq results. The qRT-PCR-based gene expression validation results showed that *Anth* was downregulated as compared with other selected genes such as *PU*, *Eth-RA*, *AbAc*, and *1-AM*. The susceptible treated and control samples showed higher levels for upregulated *PU* than for the other three selected genes, but *Eth-RA*, *Ab Ac*, and *1-AM* were slightly overexpressed as compared with *PU* in resistant treated samples. This finding might explain the resistant character of Kufri Swarna (Figure 10). 

## 3. Discussion

Plants have complex multi-layered defense mechanisms against PPNs. This defense response is called nematode-associated molecular patterns involved in recognizing and perceiving a pathogen and subsequent activation of various protection strategies that suppress infestation locally or tissues at a distance via systemic defense signaling. Similarly, the effector gene from PPNs during the host interaction can also modify different signaling pathways in hosts [37]. Considering this background information, the present investigation aimed at a comparative analysis of DEGs in response to PCN in potato cultivars and the pathways involved in resistant and susceptible mechanisms against PCN.

Gene expression profiling in common bean (*Phaseolus vulgaris* L.) revealed differential expression of several genes encoding nucleotide-binding site leucine-rich repeat resistance proteins (NLRs), WRKY TFs, pathogenesis-related (PR) proteins, and heat shock proteins (HSPs) in roots upon infection with the soybean cyst nematode *Heterodera glycines* [38]. Similarly, a recent transcriptome study of resistant and susceptible upland cotton (*Gossypium hirsutum* L.) infected with Southern root-knot nematode (*Meloidogyne incognita*) revealed the upregulation of several genes in the resistant genotype and downregulation in the susceptible genotype [39]. Genes with TF activity, defense response, cell wall organization, response to phyto-hormones, and protein serine/threonine kinase activity were upregulated in the resistant versus susceptible genotypes [39]. Another study suggested that physical reinforcement of cell walls with lignin is an important defense response against nematodes. The *Solanum torvum*–root-knot nematode system can provide a molecular basis for understanding plant–nematode interactions [40]. In *S. torvum*, a resistant root stock for eggplant and root-knot nematode interactions, infestation by an avirulent pathotype of *Meloidogyne arenaria* induced the expression of several genes such as class III peroxidases, fatty acid desaturases, sesquiterpene synthases, and genes involved in defense, hormone signaling, biosynthesis of lignin, etc., which contribute to resistance. However, infection with the virulent pathotype of *M. arenaria* induced the expression of genes that are helpful in forming a feeding site for the nematode; these genes include chalcone synthase, spermidine synthase, and genes related to cell wall modification and transmembrane transport.

Plant hormones are tuners of plant defense responses to abiotic and biotic stresses and are involved in various complex networks by which they control responses to different environmental stimuli [41]. In the present study, numerous plant hormone synthesis genes putatively involved in preventing disease development were differentially expressed in the roots of both potato cultivar genotypes in response to PCN infestation. The upregulation of the hormonal pathway associated with jasmonic acid (JA) (XP 006351473, XP 006347422) was found only in resistant Kufri Swarna. Concurrently, JA (XP 006360347)-associated genes were downregulated in susceptible Kufri Jyoti. Consistent with our results, JA-mediated defense mechanisms have been reported in various agricultural plants during host–nematode interactions [42,43,44,45,46]. In susceptible Kufri Jyoti, JA biosynthesis-related genes were suppressed by PCN infestation. The downregulation of this particular pathway can suppress the plant defense system for greater susceptibility to PCN infestation. The same results were observed in different host plants interacting with PPNs [47,48,49].

MAPKs associated with pathogen-associated molecular pattern types of plant immunity initiate hormonal defense signaling pathways via salicylic acid and JA [50]. Several MAPK signaling genes were upregulated in wild soybean interacting with *H. glycines* infestation, *Arabidopsis* with *H. schachtii* infestation, soybean with *H. glycines* infestation, and rice with root-knot nematode infestation during resistance mechanisms [51,52,53,54]. We found similar results with the resistant versus susceptible potato cultivars. 

Protein inhibitors are a range of proteinase classes widely expressed in plants in which they are often induced by wounding. The genes of protein inhibitors are also expressed tissue- and genotype-dependently after wounding and nematode invasion [55]. Cystatins have been used to protect various plant species, including alfalfa, banana, rice, and tomato, against a wide range of nematodes with diverse feeding strategies [56,57,58]. A previous study [31] revealed that cysteine protease was resistant to PCN interacting with the Swedish potato cultivar, and the interaction of *G. rostochiensis* infestation with tomato showed some protein inhibitor expression [59]. The results of this study agree with previous studies in that two cysteine proteinase inhibitor genes were found to be upregulated in the susceptible cultivar, and one such gene was found in the resistant cultivar. 

TFs seem to be involved in regulating and initiating various biological activities during host plant–nematode interactions [60]. Therefore, the upregulation of TFs in both genotypes may be due to PCN infestation stress and activating different signal transduction pathways. The functional involvement of TFs was studied in the interaction of peanut and *M. arenaria* infestation and tomato and *G. rostochiensis* infestation [61,62]. Auxin response TFs are involved in compatible plant–nematode interactions, regulating ROS activity and increased Ca^2+^ conductance across the cell membrane, as well as activating auxin-mediated defense signaling against PPNs [63]. In this study, the upregulation of auxin response TFs was found only in resistant Kufri Swarna. Similar results were found in resistant soybean interacting with *H. glycines* infestation [64].

Cell-wall-associated genes participate in the loosening, biogenesis/degradation, differentiation, organization, and proliferation of cells. Such genes are expressed during the initiation of nematode feeding sites and successful invasion into host plants [65]. The upregulation of cell-wall-associated genes clearly indicates that PCN established successful parasitism in susceptible Kufri Jyoti. Our results are consistent with those of previous studies. The alteration of cell-wall-degrading or -modifying enzymes due to root-knot nematode infestation was also found in tomato [26]. 

A few studies showed that the plant cell wall organization is altered in response to PCN infestation [66,67,68,69]. In susceptible Kufri Jyoti, two genes related to cell-wall-degrading or -modifying enzymes were downregulated. Similar results were obtained in soybean in response to soybean cyst nematode infestation [70]. Additionally, the content of glucosinolates, phenolics, or terpenoids was previously found to be increased via flavonoid and steroid biosynthesis. These genes were expressed in plant roots after successful penetration of nematodes and caused severe damage in infected plants [71,72]. In our study, the genes involved in flavonoid and steroid biosynthesis pathways were expressed in susceptible (six genes) and resistant (one gene) cultivars after PCN infestation. This finding is related to PCNs establishing a successful infestation and damaging cells in susceptible Kufri Jyoti. The plant host and the PPN itself can initiate the flavonoid biosynthesis pathway during PPN pathogenesis directly or indirectly; for example, PCN infestation (*G. pallida* and *G. rostochiensis*) modified plant flavonoid components such as quercetin and kaempferol to convert to quercentagetin, which acts as a reversible inhibitor of the plant defense mechanism (nematode unique flavonoid) [73,74]. The larvae of *M. incognita* and *Pratylenchus penetrans* never penetrated the resistant variety of tomato because of the inhibition provided by phenolic compounds [75].

Programmed cell death and the hypersensitive response related to plant defense mechanism against PPNs results from ROS signaling in plants [37]. From the RNA-seq analysis, in response to invading PCNs, plant resistance/susceptibility (R/S) elicits a series of defense mechanisms by coordinating different signaling pathways. These results agree with previous studies of plant–nematode interaction with resistant and susceptible interactions [26,31,46,76]. They are also in line with fast transcriptional reprogramming similar to the pathogen defense system, possibly because some R genes in the resistant Kufri Swarna may activate complex defense mechanisms and become resistant to PCN infestation. Rhg1 and DELLA were present in soybean showing enhanced resistance to *H. glycines* infestation (soybean cyst nematode) by activating ROS-related genes [64,77]. Of note, *Rhg1* (Soltu.DM.06G010350) expression was 1.2-fold increased in resistant Kufri Swarna but was downregulated in susceptible Kufri Jyoti. Similarly, plant pathogen-related genes were expressed in response to PCN infestation in tomato due to a HERO-A gene-based resistance response in a resistant potato line; in susceptible tomato lines, these genes were inhibited [78]. Related results were found in a wheat line resistant to *H. avenae* infestation (cereal cyst nematode) that expressed the *Cre2* gene [79].

Although defense-related proteins and plant hormones were expressed in the susceptible genotype, the susceptibility was ultimately due to their expression pattern and suppression of the host defense by PCN effectors secreted by nematode oesophageal glands during the susceptible interaction. According to Chen et al. [80], *G. rostochiensis* can promote successful plant parasitism by suppressing the plant’s defense via the suppression of plant immunity and could further generate within root tissue, the feeding cells essential for nematode development. During the interaction, the overexpressed peroxiredoxin-family genes in *G. rostochiensis* produced a series of redox reactions against the defense responses in potato [81]. The effector genes *19C07* (*H. schachtii*) and *MjCM-1* (*M. javanica*) alter auxin influx, along with chorismate-derived metabolites, lead to cell enlargement and the formation of syncytia in host plants [63,82,83].

## 4. Materials and Methods

### 4.1. In Vitro Propagation of PCN-Resistant and -Susceptible Cultivars

One-week-old sprouts of the PCN-resistant potato cultivar Kufri Swarna and the PCN-susceptible potato cultivar Kufri Jyoti were used as initial explants for in vitro micropropagation. The explants (1.0–1.5 cm in length) were surface-sterilized by washing under tap water for 10–15 min. Explants were surface-sterilized with 0.1% HgCl_2_ solution for 3–5 min, then treated with two drops of Tween-20 (wetting agent) for 5–6 min and washed several times with autoclaved distilled water. B5 medium [84] was used for nodal and inter-nodal segment culture [84]. For plant growth, we used basal nutrient salts, which contain macro- and micronutrients and vitamins. During cultivation, the pH of the medium was adjusted to 5.8 by using 1N NaOH. The medium was autoclaved at 121 °C for 20 min after adjusting the pH. Explants were inoculated in liquid medium at 18–22 °C for 3–6 weeks. The photoperiod was generally maintained at 16 h light/8 h dark for further multiplication. After regeneration, 3- to 6-week-old microplants were transferred to pots filled with sterilized coco peat, soil, and farmyard manure (3:2:1). Before planting into pots, the microplate roots were cut just above the medium and the cut end was dipped in fungicide (Diathane, 2.5 mL/L). After transplanting, pots were transferred to the greenhouse for 10 days at 27 °C.

### 4.2. PCN Hatching and Inoculation

To hatch juvenile PCNs, cysts were soaked in distilled water for 3–5 days to hydration, then the water was replaced with potato root diffusates as described [85]. After establishing plantlet pots (30 days of planting), 200 hatched second-stage PCN juveniles were inoculated near the root zone. After 14 days post-inoculation (dpi), treated and untreated Kufri Swarna and Kufri Jyoti plantlet roots were used for RNA-seq. 

### 4.3. RNA Isolation, Library Preparation, and RNA-seq

Total RNA was isolated from the root tissues of the PCN-resistant Kufri Swarna and PCN-susceptible Kufri Jyoti cultivars by using the TRIzol method. The integrity and quality of RNA were analyzed using a Qubit fluorometer. Equimolar concentrations of RNA from three biological replicates were pooled to construct an Illumina NextSeq PE library. Two 75 bp pair-end libraries for resistant Kufri Swarna and susceptible Kufri Jyoti were constructed by using the Illumina Truseq stranded mRNA Library Prep Kit following the manufacturer’s instructions. The libraries were sequenced on a NextSeq500 instrument with 2 × 75 chemistry. The resulting image files in the bcl format were converted to FASTQ with 2 × 75 bp reads using the bcl2fastq tool (Illumina, CA, USA). The raw read sequences were deposited in the Short Read Archive database at the U.S. National Center for Biotechnology Information (NCBI) (BioProject accession no. PRJNA488526).

### 4.4. Preprocessing and Genome Mapping

The quality of raw reads was ascertained by checking the adapter, G.C. distribution, average base content, and quality score of the distribution using fastqc (https://www.bioinformatics.babraham.ac.uk/projects/fastqc/, accessed on 3 February 2022). The sequencing adapters and low-quality reads (Phred score Q.V. < 30) were filtered by using the read trimming tool Trimmomatic v.0.39. The quality-filtered reads were mapped to the *Solanum tuberosum* v6.1 genome (Pham et al., 2020; https://phytozome-next.jgi.doe.gov/info/Stuberosum_v6_1, accessed on 3 February 2022) by using the STAR universal RNA-Seq alignment tool [86] with default parameters to generate BAM alignment. The read count tables for the genes across all the samples were created by using BAM alignment and the general feature format of genome annotation with HTSeq v.0.13.5 [87]. The counts were normalized by using the Trimmed Mean of M-values (TMM). The gene expression based on the read counts was studied by using the fragments per kilobase of transcripts per million (FPKM). The FPKM value for each gene was calculated according to the read count table, the total number of reads per sample, and the gene length in kilobytes.

### 4.5. Identification of Differentially Expressed Genes (DEGs)

The DEGs resulting from comparing the resistant and susceptible cultivars were identified by using the NOISeq R/Bioc package [88] with three simulated replicates, variability of 0.02, and a counts-per-million value of 1. The DEGs were filtered on the basis of a minimum log2 fold change value of 1 and *p*-value of 0.9 as per the NOISeq R/Bioc package. Gene annotation, via Gene Ontology (G.O.) enrichment analysis, was performed using Omicsbox (https://www.biobam.com/omicsbox/, accessed on 3 February 2022). Transcription factor (TF) prediction and TF enrichment analysis involved using the Plant Transcription Factor Database (http://planttfdb.cbi.pku.edu.cn/, accessed on 3 February 2022). Heatmaps were generated by using mev (http://mev.tm4.org/, accessed on 3 February 2022). Gene network analysis involved using Cytoscape (https://cytoscape.org/, accessed on 3 February 2022) and the STRING database (https://string-db.org/, accessed on 3 February 2022) with Arabidopsis as the reference to retrieve protein–protein interactions. Functional networks for DEGs were derived by using the ClueGO plugin in Cytoscape. Pathway mapping involved using KOBAS and MapMan (https://mapman.gabipd.org/, accessed on 3 February 2022). Disease resistance genes were identified in the plant disease-resistance gene database [89].

### 4.6. qRT-PCR Validation

To validate the differential expression patterns observed in the RNA-seq data, five DEGs were randomly selected for qRT-PCR investigation. cDNA was synthesized using the RT2 First Strand Kit (Qiagen, Hilden, Germany) following the manufacturer’s instructions. The eF1α gene from potato was used as an internal control. The StepOnePlus Real-Time PCR System was used for qRT-PCR analysis. The qRT-PCR validation of the five genes was performed in triplicate using KAPA SYBRR FAST qPCR Master Mix (2X) Universal (KAPA Biosystems, Wilmington, MA, USA) following the manufacturer’s protocol. The expression profiles of all unigenes were analyzed via the 2^−ΔΔCT^ method [90].

## 5. Conclusions

To better understand the molecular mechanism of plant resistance against PCN infestation, we used RNA-seq to identify differences in gene expression between infested and non-infested susceptible and resistant potato cultivars under PCN infestation. We found a series of plant defense mechanisms elucidated by the synthesis of hormones and pathogen-related proteins. Furthermore, MAPK-associated plant immunity was triggered only in resistant Kufri Swarna. Numerous effective genes associated with plant defense or hormones were arrested by PCN effectors in susceptible Kufri Jyoti. The finding of genes involved in the potato response to PCN infestation provides basic groundwork for identifying the functional genes involved in the susceptibility/resistance mechanism in potato cultivars. Further functional-based gene characterization is important to reveal the conclusive molecular mechanism and to also identify plant resistance genes for efficient use in crop improvement programs against PCN that are economically sustainable and ecologically safe. This is the first study to identify the underlying resistance against PCN and host interaction in Indian potato varieties.

## Figures and Tables

**Table 1:** Summary of RNA-seq and genome mapping from resistant (R) and susceptible (S) potato cultivars.

Particulars	Control_S	Treated_S	Control_R	Treated_R
Total number of raw reads	20,099,283	20,535,765	21,367,409	24,128,453
Total number of valid paired-end reads	19,116,371	19,544,076	19,976,274	23,014,160
Read length	75	75	75	75
GC content (%)	42	42	42	42
Q30 (%)	95.1	95.2	93.5	95.4
% of mapped reads	97.3	96.4	86.1	88.9
% of unmapped reads	2.7	3.6	13.92	11.1

## Data Availability

The raw read sequences were deposited in the Sequence Read Archive Database at the National Center for Biotechnology Information (NCBI) under the BioProject accession No. PRJNA488526.

## Supplementary Materials

The following supporting information can be downloaded at: https://www.mdpi.com/article/10.3390/plants11081008/s1, Figure S1: Life cycle of potato cyst nematode.
