# Supplementary figures and images for: RNA-Seq of Cyst Nematode Infestation of Potato (Solanum tuberosum L.): A Comparative Transcriptome Analysis of Resistant and Susceptible Cultivars
Source: Plants (Basel). 2022 Apr 7;11(8):1008. doi: 10.3390/plants11081008 (PMC9025382; doi:10.3390/plants11081008)

## Supplementary Data and Figures

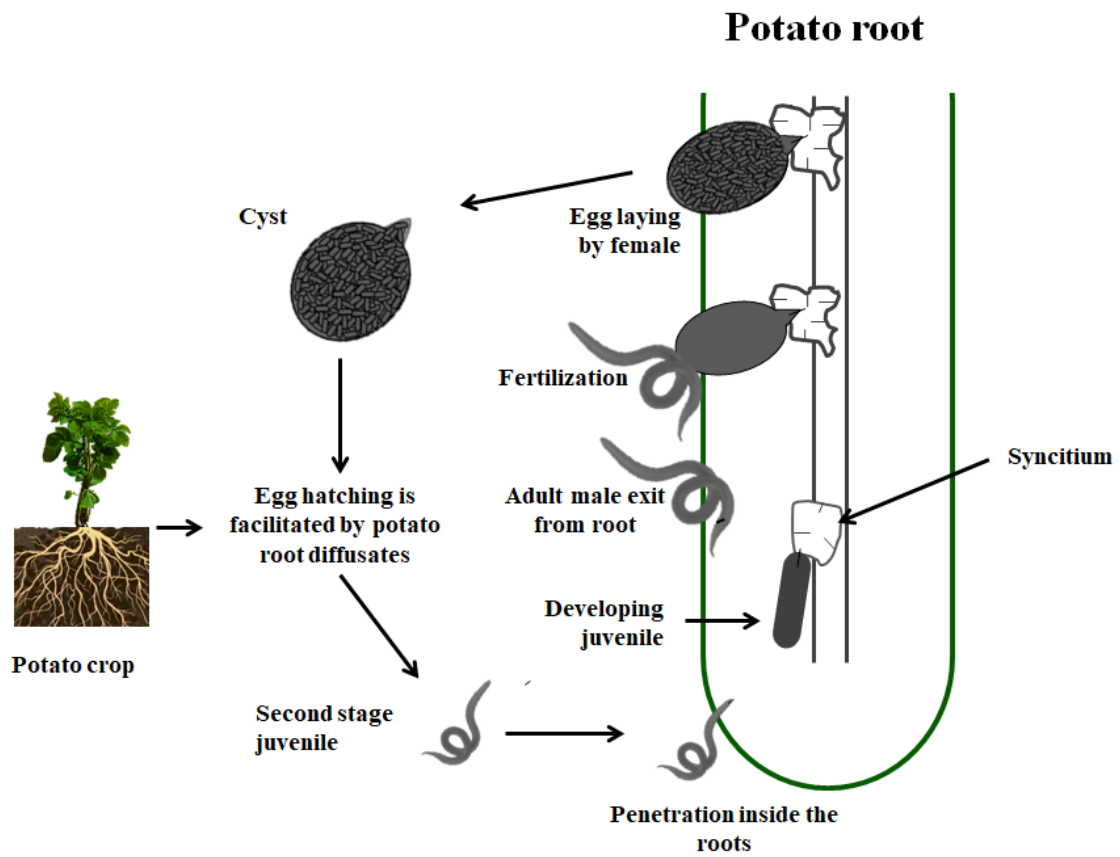

**Figure S1.** Life cycle of potato cyst nematode.

Supplement: Supplementary file 1 [file plants-11-01008-s001.zip › plants-1604768-supplementary.pdf]
